# Decreased Seizure Threshold in an Eclampsia-Like Model Induced in Pregnant Rats with Lipopolysaccharide and Pentylenetetrazol Treatments

**DOI:** 10.1371/journal.pone.0089333

**Published:** 2014-02-20

**Authors:** Qian Huang, Lei Liu, Bihui Hu, Xiaodan Di, Shaun Patrick Brennecke, Huishu Liu

**Affiliations:** 1 Department of Obstetrics, Guangzhou Women and Children’s Medical Center, Guangzhou Medical University, Guangzhou, China; 2 School of Medicine, Jinan University, Guangzhou, China; 3 Department Perinatal Medicine Pregnancy Research Centre and University of Melbourne Department of Obstetrics and Gynaecology, Royal Women’s Hospital, Parkville, Victoria, Australia; Medical Faculty, Otto-von-Guericke University Magdeburg, Medical Faculty, Germany

## Abstract

**Objective:**

Eclampsia is a poorly understood but potentially fatal complication of pregnancy. Research to date on this disorder has been hampered by the lack of a suitable animal model. To correct this deficiency, this report describes the generation of a rat eclampsia-like model using pentylenetetrazol (PTZ) in a previously established rat preeclampsia model.

**Method:**

Rats were administered lipopolysaccharide (1.0 µg/kg) by tail vein injection on gestational day 14 to establish preeclampsia (PE). PE and control rats (non-pregnant, NP; normal-pregnant, P) were injected intraperitoneally (i.p.) with PTZ (40 mg/kg) to induce seizures. In separate experiments, MgSO_4_ (270 mg/kg IP) was injected in advance of PTZ into PE rats to observe its effect on PTZ-induced seizures.

**Results:**

PE conditions were verified in rats after LPS administration by significantly higher blood pressure (*P*<0.01) and urinary albumin excretion (*P*<0.05), elevated sFlt-1 (*P*<0.05) and decreased PlGF serum levels (*P*<0.05), and evidence of hepatic dysfunction compared to control groups. PTZ successfully induced seizure activity in all groups studied. Latency to seizure was significantly (*P*<0.01) less in the PE-PTZ group (73.2±6.6 sec.) than in PTZ-treated controls (107.0±7.4 sec.). Pretreatment with MgSO_4_ prolonged (*P*<0.05) latency to seizure, shortened seizure duration and decreased seizure rates. Significant increased (*P*<0.05) in the serum levels of the inflammatory cytokines TNF-α and IL-1β in PE and PE-PTZ groups, and decreased (*P*<0.05) in their levels following MgSO_4_ administration.

**Conclusion:**

This PTZ-induced eclampsia-like rat model is comparable to the human condition of eclampsia and may serve as a useful research tool for future studies of this disease. The increased inflammatory cytokines in preeclampsia are coincident with a decreased threshold for PTZ-induced seizures, suggesting that an inflammatory mechanism may contribute to the susceptibility to seizure activity and inflammation might have an important role in eclampsia.

## Introduction

Eclampsia is characterized by grand mal seizures that cannot be attributed to other causes in women with the human pregnancy-specific disorder preeclampsia. The clinical manifestations of maternal preeclampsia are hypertension and proteinuria with or without coexisting systemic abnormalities involving the kidneys, liver, or blood. There is also a fetal manifestation of preeclampsia involving fetal growth restriction, reduced amniotic fluid, and abnormal fetal oxygenation [1]. Eclampsia manifests as one or more grand mal seizures. Worldwide, eclampsia is estimated to complicate 1.4% of all deliveries [2]. In developed countries, maternal deaths from eclampsia are rare, but in developing countries the mortality rate can be as high as 15% [3].

Although described by Hippocrates around 400 BCE [4], the pathogenesis of eclampsia remains poorly understood. This is partly because no animal model of eclampsia has been developed to date to facilitate the study of this disorder. Such a model could increase understanding of the pathophysiology of eclampsia and contribute to the development of novel therapeutics for its management.

In contrast, a number of animal models of preeclampsia have been developed and successfully used to explore aspects of this disease. The first animal model of preeclampsia was developed by ultra low dose endotoxin infusion in pregnant rats [5]. Since then, other methods such as reduced uterine perfusion pressure, induction of uteroplacental ischemia and some pharmacological treatments have also been found to mimic key features of the disease such as hypertension and proteinuria [6]–[8]. As well, data derived from such animal models as well as from human studies has demonstrated an important role for inflammatory mediators and angiogenic modulators in the pathogenesis of preeclampsia [9]–[11].

The aim of this study therefore was to use an established animal model of preeclampsia to develop an animal model of eclampsia to enable future studies on the pathogenesis and management of eclampsia. To this end, an endotoxin infusion rat preeclampsia model was treated with the epileptogenic agent pentylenetetrazol (PTZ) to create an eclamptic model. This model and its response to the clinically relevant anticonvulsant drug magnesium sulphate (MgSO_4_) were then characterized in terms of various seizure, biometric and biochemical parameters to evaluate its potential utility as a research tool.

## Materials and Methods

### Animals

This study was carried out in strict accordance with the recommendations in the Guide for the Care and Use of Laboratory Animals. The protocol was approved by the Committee on the Ethics of Animal Experiments of the Guangzhou Medical University (Permit Number: 2012–50). All surgery was performed under chloral hydrate anesthesia, and all efforts were made to minimize suffering. The Medical Experimental Animal Center of Guangdong, China, provided the 57 female Sprague-Dawley rats (200–240 g, aged 2 months) for this study. The animals were housed under controlled conditions (23–26°C, relative humidity 50–60%, illumination between 6∶00 am and 6∶00 pm) and had free access to water and pellet food. The rats were allowed one week of animal house acclimatisation before experiments were commenced.

### Reagents

Lipopolysaccharide endotoxin (LPS; *E. Coli* 0.55:B5, Sigma Aldrich, USA), PTZ and MgSO_4_ (Sigma Aldrich, USA) were dissolved in sterile pyrogen-free 0.9% saline. A stock solution of 1 mg/ml LPS was frozen in aliquots, which were thawed to prepare fresh drug solutions for administration when needed. A 1% (g/100 ml) PTZ solution was freshly prepared each day. A 10% MgSO_4_ solution was prepared and stored at 4°C. ELISA kits specific for tumor necrosis factor-α (TNF-α) and interleukin-1β (IL-1β) were obtained from eBioscience (San Diego, USA), those for placenta growth factor (PlGF) and soluble FMS-like tyrosine kinase-1 (sFlt-1) were obtained from R&D Systems (Minnesota, USA).

### Study Design

**Figure 1:**
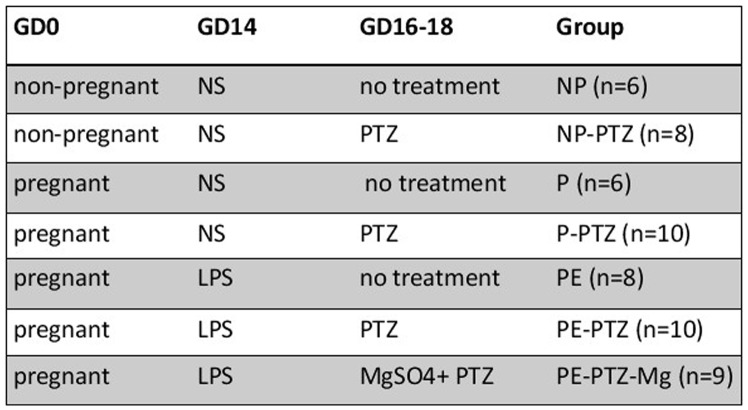
Study design. 57 Sprague-Dawley rats were randomly divided into seven groups designated: On gestational day 14, LPS was given to the rats comprising the preeclampsia model, while rats in the control group were given normal saline. 40 mg/kg PTZ was given on GD16–18 (i.p.) to induce seizure. Magnesium sulfate (MgSO_4_) was given 20 minutes prior to receiving PTZ. NP = non-pregnant control (n  = 6); NP-PTZ = non-pregnant rats+PTZ (n  = 8); P = normal-pregnant control (n  = 6); P-PTZ = normal-pregnant+PTZ (n  = 10); PE = preeclampsia rats (n  = 8); PE-PTZ = preeclampsia rats+PTZ (n  = 10); PE-PTZ-Mg = preeclampsia rats+PTZ+MgSO_4_ (n  = 9). NS, normal saline.

The 57 rats used in this study were divided into groups as shown in Figure 1. To establish a preeclampsia model in pregnant rats (i.e., the PE group) we first infused an ultra-low dose (1.0 µg/kg body weight) of LPS via the tail vein in accordance with a method published previously [5]. Then, a subconvulsive dose (40 mg/kg body weight) of PTZ was given to the PE rats via intraperitoneal (i.p.) injection to induce a seizure state that mimics human eclampsia. In subsequent experiments, MgSO_4_ (270 mg/kg body weight, i.p.) was given 20 minutes before PTZ injection to observe the treatment effect on this animal model.

### Confirmation of Pregnancy

Adult female rats were housed with fertile males for one night, and then vaginal smears were taken and examined by microscopy the following morning. If sperm were found in the vaginal smear, that day was designated as gestational day 0 of the pregnancy (GD 0).

### LPS-Induced PE Model

Based on the report of Faas et al. [5], on GD 14, rats in the preeclampsia (PE) group were infused with LPS (1.0 µg/kg body weight in 2 ml of pyrogen-free saline solution) via tail vein infusion using an infusion pump at the rate of 2 ml/h. Non-pregnant (NP) and pregnant (P) control rats were administered vehicle alone under identical conditions. We used the same dose as Faas et al. [5], but adopted one-time tail vein puncture administration for LPS infusion, which is more simple to accomplish and can reduce the risk of infection, instead of inserting the cannula into the right jugular vein as done by Faas et al. [5]. Also, in contrast, we performed the infusion under anesthesia to keep the rats calm for an hour during treatment.

### PTZ-Induced Eclampsia-like Model

On GD 16, 17 and 18, rats in the each of the NP, P and PE groups were intraperitoneally injected with PTZ (40 mg/kg body weight) to induce seizures (between 5 pm. to 8 pm.). Immediately after the injection, each rat was placed in the center of a cage (30×30×50 cm), and its behavior was monitored for 30 minutes. A modified version of the seizure scale described by Racine [12], [13] was used to classify seizure severity into 5 stages as follows: stage1 - immobility, eyes closed, and facial clonus; stage 2 - head nodding and more severe facial clonus; stage 3 - clonus of one forelimb; stage 4 - rearing with bilateral forelimb clonus; and stage 5 - generalized tonic-clonic seizures. The scoring for the seizures was carried out by an observer unaware of the animal’s group. The following seizure parameters were measured: latency to stage 1 seizure, latency to stage 5 seizure, ratio of stage 5 seizure, duration of stage 1–4 seizure, and duration of stage 5 seizure.

### Effect of MgSO4 on Eclampsia-like Model

To investigate the effect of MgSO_4_ on seizures induced by PTZ in PE rats, and based on the protocol used by Cotton et al [14], [15], MgSO_4_ (270 mg/kg body weight, i.p.) was administered to PE rats 20 minutes before the injection of PTZ on GDs 16, 17 and 18, and seizure behavior as described was recorded in PE-PTZ-Mg group.

### Measurement of Biometric Parameters

Tail-cuff systolic blood pressure (SBP) was measured on GDs 6, 11, 14 (just before LPS infusion), 15, and 18 (between 9 am. to 12 am.) in the P and PE groups and on the corresponding day in the NP group. Before measurements were first undertaken, rats were exposed to the measurement process to allow their adaptation to it. Rats were also allowed to settle for 30 minutes before study BP measurements commenced. BP measurements were performed three times on each occasion for each rat, and the mean values were recorded.

On GD 19, the rats were euthanized. Pups were then removed, counted and weighed. Pup viability, resorption and gross morphology were also assessed. Placental weights were measured.

### Measurement of Biochemical Parameters

On GDs 7, 12, 16, and 19, rats were placed individually in metabolic cages for 24 h (from the previous day at 6 pm. to the recorded day at 6 pm.) to collect urine for 24 h urinary albumin excretion measurements using an automatic analyzer (HIACHI 7600-020, Japan). After rats were anesthetised with a non-lethal dose of chloral hydrate on GD 19 and blood samples collected from the inferior cava vena. The rats were then euthanized. Serum measurements of liver enzymes (ALT, AST), blood urea nitrogen (BUN), and creatinine (Cr) were performed using an automatic analyzer (HIACHI 7600-020, Japan). ELISA determinations of TNF-α, IL-1β, sFlt-1 and PlGF were performed. For each assay, standards and samples were tested in duplicate.

### Statistics

Data normal distribution was tested by the Shapiro-Wilk test. Normally distributed continuous data are expressed as mean ± standard error of the mean (SEM). Statistical analyses were performed to compare average values between different groups by analysis of variance (SPSS13.0) followed by the least significant difference (LSD) post hoc test or Dunnett’s T3 test as appropriate. Data on SBP and 24-h urinary albumin level were analyzed by one-way analysis of variance for repeated measures (analysis of variance for repeated measures). Ratios were compared using the chi-squared test or Fisher’s exact test. A *P* value <0.05 was considered statistically significant.

## Results

### LPS-Induced PE Model

A rat preeclampsia model was established which exhibited significant and characteristic hypertension, albuminuria, hepatic and placental dysfunctions and biochemical mediator abnormalities compared to control pregnant rats.

#### Systolic blood pressure

**Figure 2:**
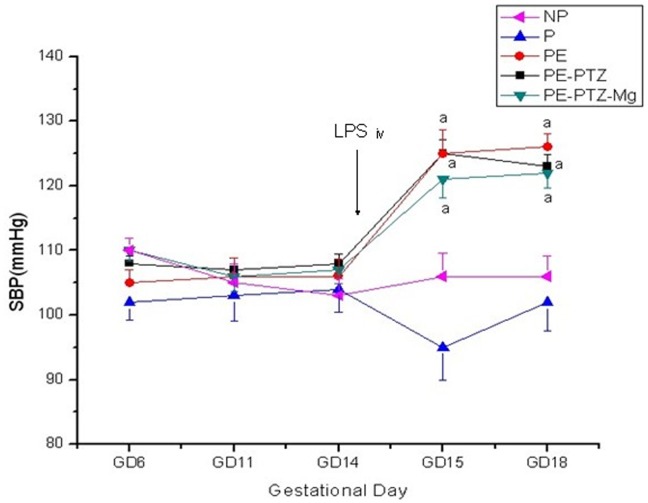
Mean systolic blood pressures of each groups measured before and after endotoxin infusion. Data are expressed as mean ± SEM of the SBP (mmHg)(repeated measures analysis of variance). NP, non-pregnant rats; P, pregnant rats; PE, preeclamptic rats. SBP was measured on GDs 6, 11, 14, 15, and 18 in pregnant groups and on corresponding days in NP group. (a) *P*<0.01, PE, PE-PTZ and PE-PTZ-Mg group on GDs 15 and 18, compared with NP and P group on the corresponding GDs and prior to the infusion of LPS on GD 14.

There was no significant difference in SBP among the groups before the infusion of LPS on GD 14. The mean SBP in the P control group did not vary significantly throughout normal pregnancy. Similarly there were no significant changes in SBP in the NP control group. After the infusion of LPS into pregnant rats on GD 14, the mean SBP in the PE group was significantly higher on GDs 15 and 18, compared with its level in the P group on the corresponding GD and prior to the infusion of LPS (*P*<0.01) (Figure 2).

#### Twenty-four-hour urinary albumin excretion

**Figure 3:**
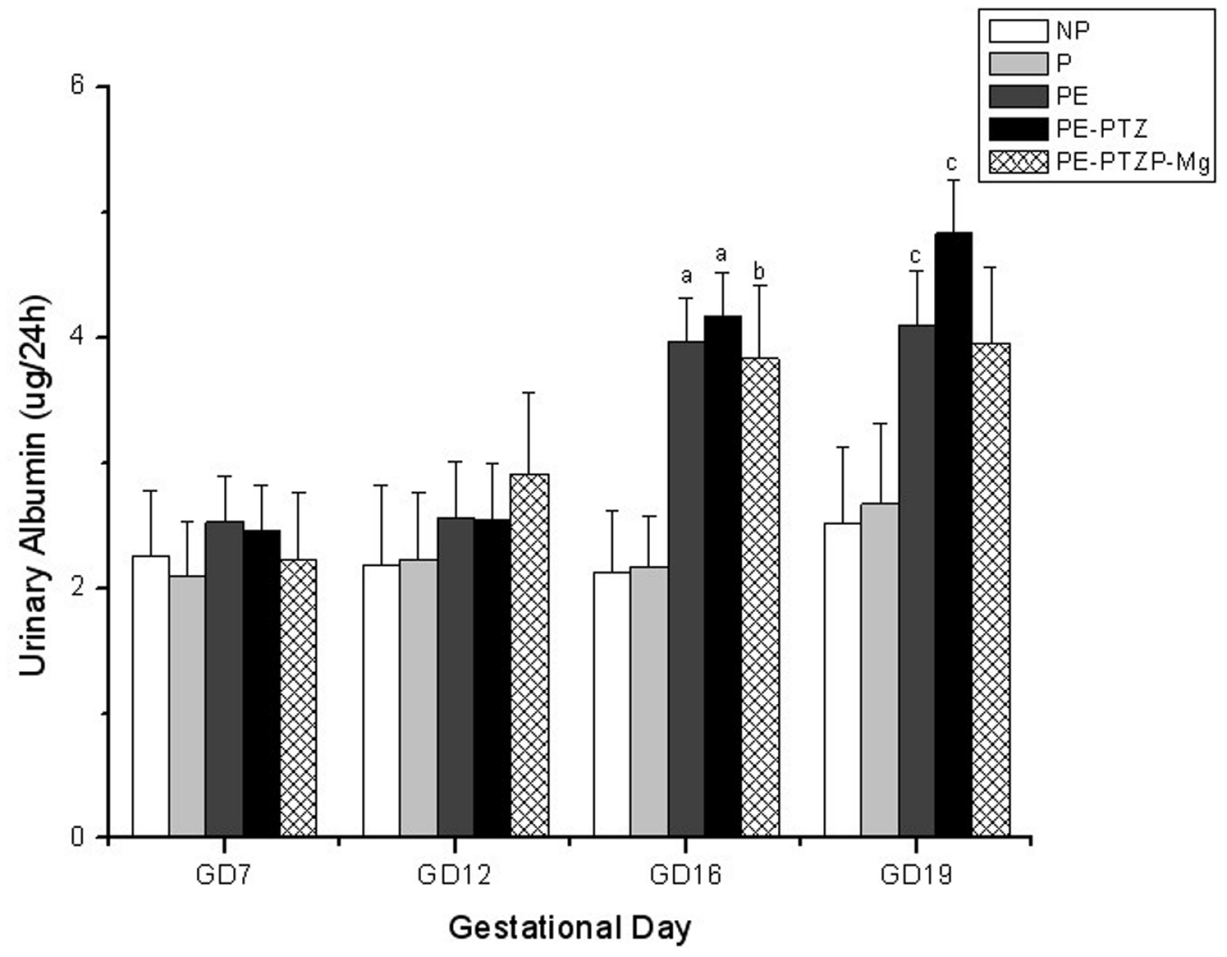
Twenty four-hour urinary albumin on different GDs. he 24-h urinary albumin was measured on GDs 7, 12, 16, and 19 using an automatic analyzer. The values are mean ± SEM of the 24-h urinary albumin in each group (repeated measures analysis of variance). (a) *P*<0.01, PE and PE-PTZ group vs. NP and P groups on GD 16. (b) *P*<0.05, PE-PTZ-Mg group vs. NP and P groups on GD 16. (c) *P*<0.05, PE and PE-PTZ group vs. NP and P groups on GD 19.

The mean 24-h urinary albumin level did not significantly change throughout the pregnancies of the rats of the NP and P group, while its levels in PE rats on GDs 16 and 19 were significantly higher than those of the NP and P group (*P*<0.05) (Figure 3).

#### Other biochemical parameters

**Figure 4:**
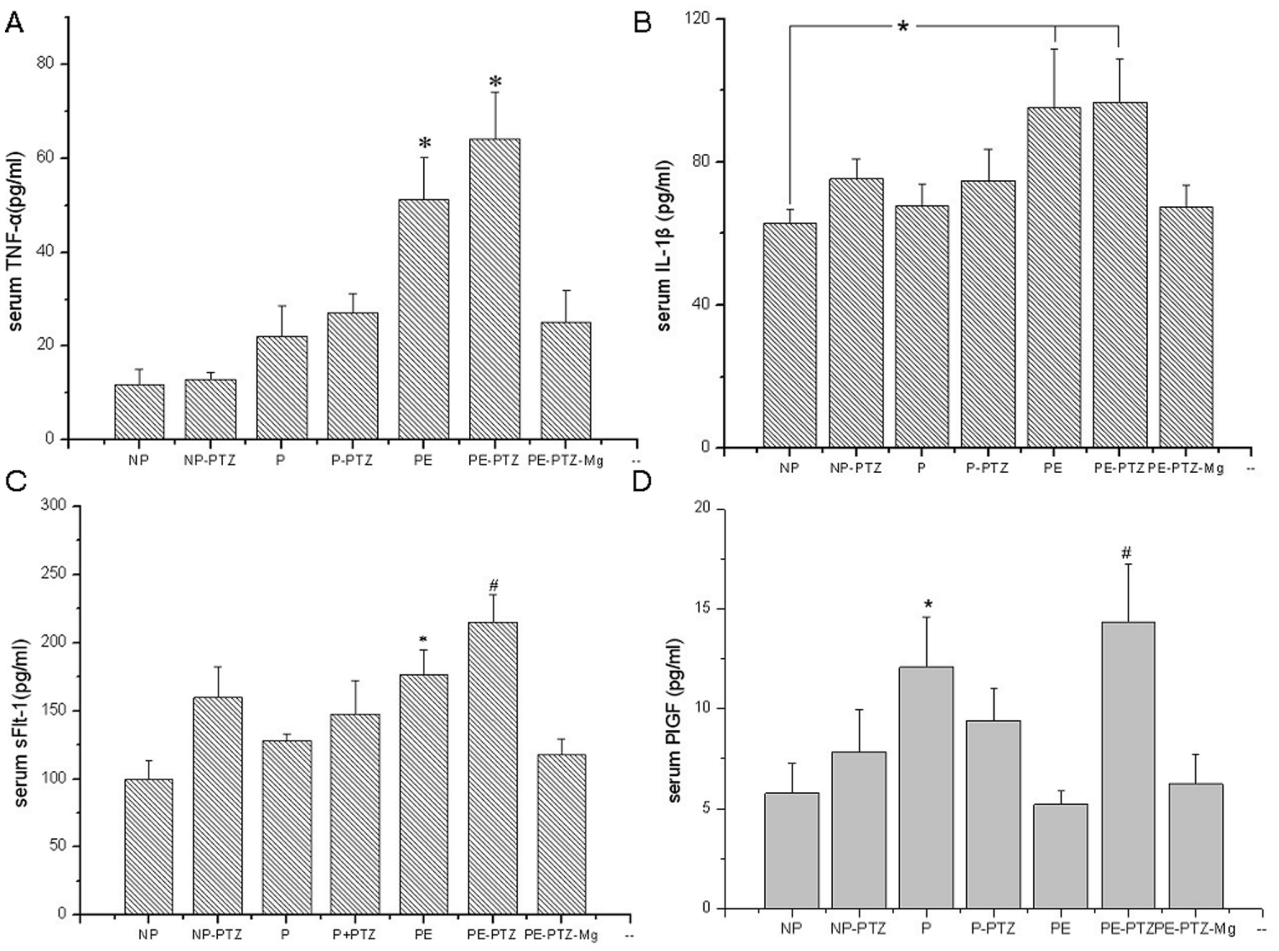
Inflammatory cytokines and preeclampsia biomarkers in different groups on GD 19. **A** * *P*<0.05, PE-PTZ and PE groups compared with NP, NP-PTZ, P, P-PTZ and PE-PTZ-Mg groups. Serum levels of TNF-α significantly increased in the PE-PTZ and PE groups, and decreased in the MgSO_4_-treated group. **B** * *P*<0.05, PE-PTZ and PE group compared to NP group. Serum levels of IL-β were higher in PE-PTZ and PE group. **C** * *P*<0.05, PE groups compared with NP and PE-PTZ-Mg groups. ^#^ *P*<0.05, PE-PTZ groups compared with NP, NP-PTZ, P, P-PTZ and PE-PTZ-Mg groups. Serum sFlt-1 increased in the PE-PTZ and PE groups. **D** * *P*<0.05, P groups compared with NP, NP-PTZ, PE and PE-PTZ-Mg groups. ^#^ *P*<0.05, PE-PTZ groups compared with NP, PE and PE-PTZ-Mg groups. Serum PlGF decreased in the PE group compared with the P group, but increased in the PE-PTZ group. (analysis of variance).

There were no significant difference in ALT, Cr, or BUN among the groups. However, AST was significantly higher in the PE group (151.1±14.1 U/L) compared with the P (122.7±7.4 U/L) and NP (113.8±5.2 U/L) control rats, which indicates liver dysfunction in the PE group (Table 1). As well, the concentrations of serum TNF-α was significantly higher in the PE group compared with the P control group (*P*<0.05), IL-1β and sFlt-1 were significantly higher when compared to the NP control group (*P*<0.05), while PlGF was significantly lower than the P group (*P*<0.05) (Figure 4).

**Table 1:** Biochemical indices of different groups on GD 19.

	n	ALT(U/L)	AST(U/L)	Cr(µmol/L)	BUN(mmol/L)
NP	6	60.5±3.7	113.8±5.2	34.5±0.8	6.0±0.4
NP-PTZ	7	76.0±8.1	130.0±13.5	34.6±2.2	7.3±0.5
P	6	73.3±2.8	122.7±7.4	31.8±3.3	6.5±0.3
P-PTZ	9	64.4±7.8	103.8±11.3	33.0±2.0	6.0±0.6
PE	8	72.9±11.3	151.1±14.1^c^	32.5±1.8	6.7±0.2
PE-PTZ	10	101.80±16.0^a^	188.1±9.6^b^	35.4±1.9	6.7±0.3
PE-PTZ-Mg	5	70.4±8.4	121.4±9.5	29.2±2.4	5.5±0.2

The serum levels of ALT, AST, Cr, and BUN were measured by an automatic analyzer. The values are mean ± SEM of each index of 5 to 10 rats in each group (in some rats there was insufficient serum for the planned analyses to be undertaken, analysis of variance). PE-PTZ-Mg, PE rats treated with 270 mg/kg MgSO_4_ and 40 mg/kg PTZ.

a *P*<0.01, PE-PTZ group compared with other groups;

b *P*<0.01, PE-PTZ group compared with other groups;

c *P*<0.05, PE group compared with other groups.

### PTZ-Induced Eclampsia-like Model

A rat eclampsia-like model was established which exhibited seizure activity in preeclamptic rats. This activity was significantly attenuated following treatment with MgSO_4_ in an analogous manner to the eclampsia clinical scenario in human pregnancy.

#### Systolic blood pressure

In the eclampsia-like model (PE-PTZ) rats, a significantly increased SBP compared to the P control group was found after induction of LPS using (*P*<0.01) (Figure 2). This increase was similar to that found in the PE group.

#### Twenty-four-hour urinary albumin excretion

LPS remained as in PE rats. The 24-h urinary albumin excretion significantly increased in the PE-PTZ group after induction of PE using LPS (*P*<0.05) (Figure 3). This increase was similar to that found in the PE group.

#### Other biochemical parameters

While the Cr and BUN measurements in the PE-PTZ group were not significantly different from those in the other groups, the ALT and AST levels in the PE-PTZ group were increased significantly compared to the other groups (*P*<0.05) (Table 1). Also, TNF-α, IL-1β, sFlt-1 and PlGF all reached their highest values in the PE -PTZ group compared to the other groups, with some but not all of the differences reaching statistical significance (*P*<0.05) (Figure 4).

#### Characterization of seizures induced by PTZ in PE rats

In rats treated with PTZ, the latency to stage 1 seizure in the PE-PTZ group (73.2±6.6 s) was significantly less than that of the P-PTZ (107.0±7.4 s) or NP-PTZ (122.5±10.4 s; *P*<0.01, Table 2). There were, however, no significant difference in the latency to stage 5 seizure, duration of stage 5 seizures, or duration of stage 1–4 seizures among the different groups. Also, there was no statistically significant difference between the stage 5 seizure ratios between the P-PTZ and the PE-PTZ groups on any of GDs 16, 17 or 18 (Table 3).

**Table 2:** The average time-course of the seizure after PTZ i.p. injection on GDs 16–18.

	n	Latency to stage 1seizure (s)	Latency to stage 5seizure (s)	Duration of stage 5seizures (s)	Duration of stage 1–4seizures (s)
NP-PTZ	8	122.5±10.4	232.5±84.9	43.1±3.4	254.8±35.8
P-PTZ	10	107.0±7.4	317.6±78.4	51.7±8.9	480.0±90.1
PE-PTZ	10	73.2±6.6^a^	275.4±35.0	41.0±3.5	587.0±175.4
PE-PTZ-Mg	9	181.5±15.4	320.0±30.4	28.3±1.7^b^	580.4±48.1

Latency and duration on each day averaged for each animal. Average time courses are expressed as mean ± SEM (analysis of variance). S = seconds.

a *P*<0.01, PE-PTZ compared with P-PTZ, NP-PTZ, and PE-PTZ-Mg.

b *P*<0.05, PE-PTZ-Mg compared with PE-PTZ.

**Table 3:** The total stage 5 seizure ratio within 30

	GD16	GD17	GD18
NP-PTZ	2/8	2/8	3/8
P-PTZ	6/10	5/10	4/10
PE-PTZ	7/10	7/10	7/10
PE-PTZ-Mg	3/9	1/9*	1/9*

*P<0.05, PE-PTZ-Mg compared with PE-PTZ group on GDs 17 and 18 indicates that MgSO_4_ treatment decreases the ratio of tonic-clonic seizure in the PE-PTZ group (Fisher’s exact test).

#### Pregnancy outcome

The percentage of resorbed fetuses was significantly higher in the PE-PTZ group compared to the other groups (*P*<0.01). The highest fetal and placental weights were observed in the P group (*P*<0.05). However, there was no significant difference in the number of live fetuses, resorptions, or malformations among the groups (Table 4).

**Table 4:** Pregnancy outcomes in different groups on GD 19.

Group (n)	Live fetuses (total)	Percent of resorbed and malformation fetuses (n)	Fetal weight (g)	Placental weight (g)
P(6)	9.3±1.1 (56)	0% (0)	4.2±0.14 ^b^	0.66±0.021 ^c^
P-PTZ(10)	8.8±0.9 (88)	8.3% (8)	3.1±0.03	0.61±0.015
PE(8)	8.5±0.8 (68)	6.8% (5)	3.0±0.04	0.61±0.014
PE-PTZ(10)	7.7±1.2 (88)	21.4% (24) ^a^	3.1±0.05	0.60±0.014
PE-PTZ-Mg(9)	10.9±0.8 (94)	4.1% (4)	3.0±0.03	0.58±0.009

The values are expressed as mean ± SEM. ^a^ *P*<0.01, the percent of resorbed fetuses is significantly higher in the PE-PTZ group compared with P, P-PTZ, and PE-PTZ-Mg groups (chi-squared test). ^b^ *P*<0.01, the fetal weight is significantly higher in the P group than in other groups (analysis of variance). ^c^ *P*<0.05, the placental weight is significantly higher in the P group than in PE-PTZ-Mg group (analysis of variance).

### Effect of MgSO4 on Eclampsia-like Model

**Seizures.** The average latency to stage 1 seizure in the PE-PTZ-Mg group was significantly longer (181.5±15.4 vs. 73.2±6.6, *P*<0.01) and the average duration of stage 5 seizure was significantly shorter (26.7±4.4 vs. 47.4±5.4, *P*<0.05) than that of the PE-PTZ rats (Table 2). In addition, in the PE-PTZ-Mg group, the stage 5 seizure ratios on GDs 17 and 18 were significantly reduced compared with that the PE-PTZ stage 5 seizure ratios on the same GDs (*P*<0.05 ) (Table 3).

#### Biochemical parameters

The SBP and 24-h urinary albumin excretion were significantly increased after LPS infusion (*P*<0.05). These increases were not significantly different in the PE-PTZ group compared to the PE-PTZ-Mg group (Figures 2 and 3). The levels of ALT, AST, TNF-α, IL-1β, sFlt-l and PIGF in the PE-PTZ-Mg group were all lower compared with those of the PE-PTZ group, with some but not all of the differences reaching statistical significance (*P*<0.05) (Table 1 and Figure 4).

#### Pregnancy outcome

Fetal and placental weights were not found to differ between the PE-PTZ-Mg and PE-PTZ groups (*P*>0.05), while the percentage of resorption of fetuses in the PE-PTZ-Mg group was significantly lower when compared with that of the PE-PTZ group (4.1% vs. 21.4%, *P*<0.01 ) (Table 4).

## Discussion

In this study, using the epileptogenic drug PTZ, we have generated and characterized an experimental model of eclampsia-like in a previously established rat model of preeclampsia.

PTZ is a γ-aminobutyric acid (GABA) receptor antagonist with high biological membrane penetrance and consequent rapid bioavailability and distribution to all organs including the brain, and very short latency of seizure induction [16]–[18]. The epileptogenic mechanism of action of PTZ is thought to involve its effects on neuronal synaptic and extrasynaptic GABA receptor function [19], [20]. PTZ-induced seizures in non-pregnant rodents have been suggested as an appropriate model for the study of human seizures and PTZ is therefore commonly used to induce seizures experimentally [21]. However, whether or not GABA receptor dysfunction is implicated in the pathogenesis of eclampsia is unknown, and so this may be a limitation of a PTZ-induced eclampsia-like model.

On the other hand, the model bears sufficient similarities to the human condition of eclampsia to suggest it may be a useful research tool. For example, in so far as pregnant women with preeclampsia are predisposed to eclampsia, the significant latency to stage 1 seizure decrease in the PE-PTZ group compared to the P-PTZ group suggests a similar predisposition to seizure activity in the rat model. As well, the effect of MgSO_4_ on the rat eclampsia-like model supports its comparability with the condition of eclampsia seen in humans. MgSO_4_ has long been used in the management of severe preeclampsia-eclampsia in clinical settings. According to large-scale population studies, MgSO_4_ administration prophylactically reduces the risk of developing eclampsia and is the anticonvulsant agent of choice in the treatment of eclamptic seizures [22]–[24]. In the current study, MgSO_4_ significantly attenuated latency, duration and rate aspects of the PTZ-induced seizure pattern and improved pregnancy outcome in terms of reduced fetal resorption rate. In so far as the occurrence of eclampsia is an indicator of the severity of preeclampsia, it is of note that the majority of the biochemical parameters monitored in this study and which reflect disease severity were highest in the PE-PTZ group. Furthermore, poor pregnancy outcome as indicated by decreased fetal and placental weights and an increased rate of fetal resorption was observed in the PE-PTZ group. In combination, these data support the experimental utility of this rat PTZ-induction model of preeclampsia. MgSO_4_ is known to be a weak antihypertensive agent [25]. In this study MgSO_4_ treatment seems to have no effect on either blood pressure or urinary albumin excretion, which is consistent with the clinical experience. Some previous studies demonstrate that MgSO_4_ prevents the development of hypertension and proteinuria [26], [27]. In comparison, most of these models were induced by N^G^-Nitro-L-arginine Methyl Ester (L-NAME) and the dose of MgSO_4_ was significantly higher or by a continual infusion method. The different induction agents, dosages and administration routes may account for these different results. Our results are in agreement with a previous study by Standley CA et al. in 2006 [28]. In this study, the MAP in a MgSO_4_ treated group did not decrease significantly until the fourth day after MgSO_4_ treatment. They found SBP significantly negatively correlated with serum ionized magnesium level on GD 21, while proteinuria did not show significant difference [28]. The data from our study further support this model which mimics the clinical human disease.

The biochemical data from this study relating to the inflammatory mediators TNF-α and IL-1β and the preeclampsia biomarkers sFlt-1 and PlGF are also worthy of note. The cytokines TNF-α and IL-1β are primarily produced by macrophages and in preeclampsia participate in abnormal extravillous trophoblast invasion [29], [30]. Tosun et al. [31] recently showed that the serum level of TNF-α was significantly increased in preeclamptic patients, especially in severe preeclampsia. IL-1β is a pivotal component of the proinflammatory response that can be harmful to the host, but the report of altered serum IL-1β levels in women with preeclampsia remains controversial [32]–[34]. In 2012, Amash et al. used an ex vivo placental perfusion system and found that preeclamptic placentas secreted higher levels of IL-1β [35]. These authors therefore suggested that the placenta may be a source of elevated maternal serum IL-1β in preeclampsia. Cipolla et al. [36] recently found that serum from pregnant rats, instead of non-pregnant ones, caused hyperexcitability to hippocampal neurons of slice culture and seizure activity that was abrogated by inhibition of TNF-α signaling. Meanwhile, application of TNF-α mimicked this increased excitability *in vitro*. Interestingly in the present study, levels of both TNF-α and IL-1β were significantly higher in the PE and PE-PTZ groups. Such an increase in inflammatory cytokines not only supports the concept of inflammatory mechanisms contributing to the pathogenesis of preeclampsia [10], [30], but also raises the possibility that such an increase may be contributory to the susceptibility to seizure activity *in vivo* in the PE-PTZ group. The significantly decreased TNF-α level in the PE-PTZ-Mg group compared to the PE-PTZ group is also consistent with this idea. In addition, MgSO_4_ administration is known to have anti-inflammatory effects [37], [38]. However, the lack of blood pressure response to MgSO_4_ even after reduction of these factors suggests that the hypertension in this model is mediated by addition factors.

High serum levels of sFlt-1 and low serum levels of PlGF have been proposed as useful predictors for the subsequent development of preeclampsia. Pro-angiogenic PlGF is inactivated by interaction with sFlt-l, thus reducing the free PlGF [39], [40]. Levine et al. [41] reported that sFlt-1 tended to increase about 5 weeks before the onset of preeclampsia. PlGF levels were significantly lower as early as 13–16 weeks gestation in women who later become preeclamptic compared with controls, but the greatest difference occurred during the onset of preeclampsia, coincident with an increase in the sFlt-1 level [41], [42]. In the present study, a similar increase in sFlt-1 and decrease in PlGF were seen in the PE group compared to the P control group. However, although sFlt was also elevated as expected in the PE-PTZ group, PlGF in this group was paradoxically not decreased. By way of explanation of this result, it has been shown previously by Xu et al. [43] in a case control study that PlGF is elevated in the cerebral spinal fluid of epileptic patients compared with non-seizure subjects. This suggests that PlGF may be increased as a consequence of seizure activity. This hypothesis supported by the finding in this study of a significant reduction in serum PlGF in the PE-PTZ-Mg group compared to the PE-PTZ group.

In conclusion, this report describes a rat model of eclampsia-like with a number of clinical, biochemical and pharmacological features that make it comparable to the human disease and therefore a useful research tool for further studies on the pathophysiology and clinical management of this important and poorly understood obstetric condition. An increase in the serum levels of the inflammatory cytokines and changes in their levels following MgSO_4_ administration suggest an inflammatory mechanism may contribute to the susceptibility to seizure activity in preeclampsia rats.
